# Vocal cord palsy after vincristine treatment in a child and the inefficacy of glutamic acid in the prevention of relapse: a case report

**DOI:** 10.1186/1752-1947-6-128

**Published:** 2012-05-14

**Authors:** Piero Farruggia, Serena Tropia, Sonia Cannella, Giuseppa Bruno, Gaspare Oddo, Paolo D’Angelo

**Affiliations:** 1 Oncology Department, Pediatric Hematology and Oncology Unit, A.R.N.A.S. Ospedali Civico, Di Cristina e Benfratelli, Piazza N. Leotta 4, Palermo, 90127, Italy; 2 Clinic Department, Pharmaceutical Laboratory, A.R.N.A.S. Ospedali Civico, Di Cristina e Benfratelli, Piazza N. Leotta 4, Palermo, 90127, Italy

**Keywords:** Glutamic acid, Neuropathy, Vincristine, Vocal cord palsy

## Abstract

**Introduction:**

Vincristine is an antineoplastic drug with a well known efficacy for the treatment of acute lymphoblastic leukemia and many solid tumors. No more than 20 pediatric patients with vincristine-induced vocal cord palsy have been reported, and to the best of our knowledge this is the first case where glutamic acid was administered with the aim of preventing a relapse of laryngeal dysfunction.

**Case presentation:**

The larynx paralysis presented with hoarseness and stridor in a Caucasian 18-month-old girl and spontaneously resolved in about a month. In order to administer a subsequent full dose of vincristine, our patient received oral glutamic acid whose efficacy against vincristine neurological side effects has been previously reported.

**Conclusions:**

Since in our patient the amino acid proved to be ineffective in the prevention of laryngeal paralysis relapse, we suggest that a dose reduction of vincristine should be preferred by oncologists as an initial approach after a case of drug-induced vocal cord palsy.

## Introduction

Vinca alkaloids have a proven role in the treatment of hematological neoplasms. The basis of their action as mitotic inhibitors is binding to microtubule proteins, but they may cause axonal degeneration. In some cases, it is likely a role in the onset of neurotoxicity can be related to pre-existing liver dysfunction or concomitant use of drugs such as itraconazole, phenytoin, isoniazid, erythromycin, azoles or allopurinol. Rarely, vincristine (VCR) can produce vocal cord palsy (VCP) as a consequence of peripheral neurotoxicity involving cranial nerves, a potentially life-threatening event.

## Case presentation

**Figure 1:**
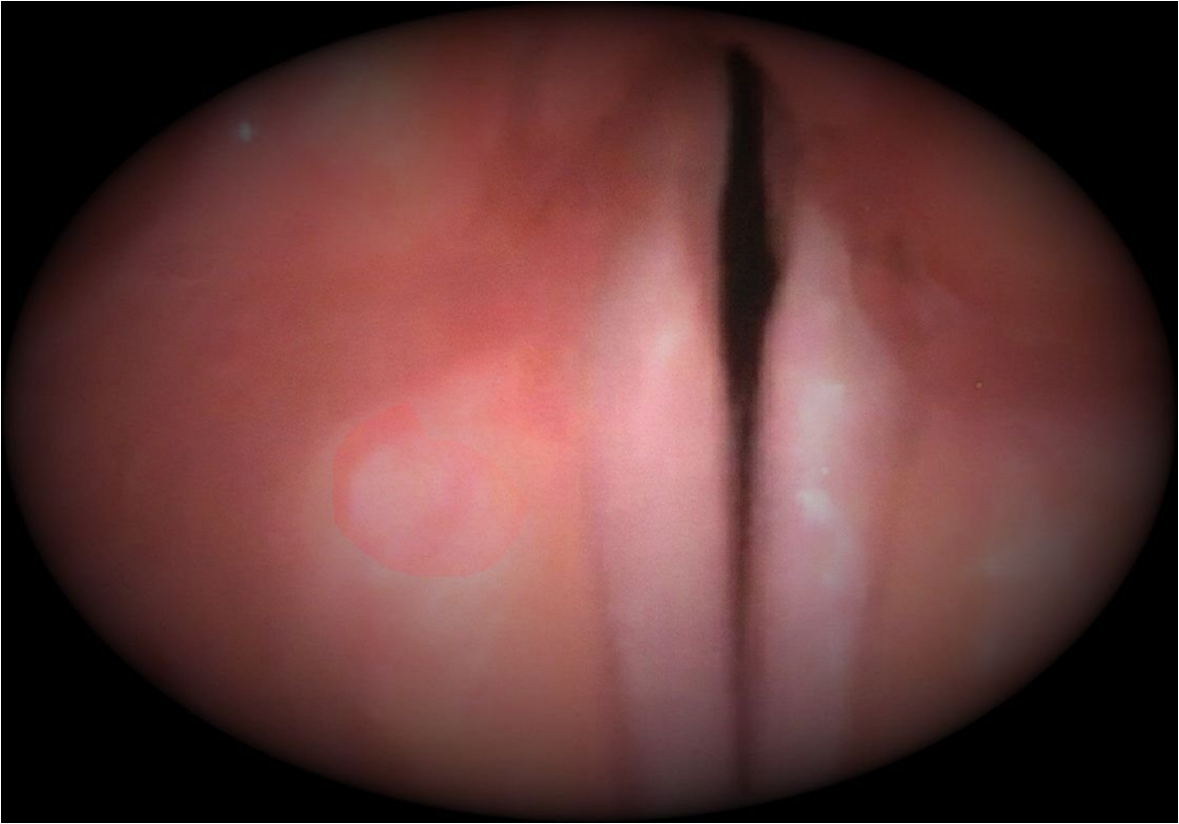
Vocal cord palsy: endoscopic view.

A Caucasian 18-month-old girl, born to an Italian family and raised in Italy, with a high-risk acute lymphoblastic leukemia (ALL) was being treated according to the AIEOP-BFM ALL 2000 protocol. After the third dose of VCR (1.5 mg/m^2^) she developed isolated hoarseness, but stridor appeared soon after the fourth dose of VCR (last dose of induction phase). A flexible fiber-optic endoscope study showed a bilateral VCP (Figure 1). Electromyography revealed a predominantly axonal motor neuropathy involving above the lower extremities. A nerve conduction study of the larynx was not performed because the association of VCR administration with significant respiratory symptoms and immobility of the vocal folds, clearly shown by the fiber-optic endoscope, allowed us to make a definite diagnosis. No side effects of VCR other than VCP were found. Her stridor started to improve within the first seven days and both stridor and hoarseness completely resolved 28 days after the onset of palsy: a repeat laryngoscopy study showed normal vocal cord mobility.

Following two consolidation blocks it was decided to administer VCR at full dose (1.5 mg/m^2^) first, and completely omit the second dose (scheduled just five days later): no side effect was noted. The first reinduction protocol II started 22 days after completion of consolidation block therapy and the four weekly doses of VCR were uneventfully administered with a one-third reduction (1 mg/m^2^).

After four weeks of interim maintenance the second reinduction protocol II was scheduled and the first weekly dose of VCR was again administered at 1 mg/m^2^ without any problems; then our patient received glutamic acid 1.5 g daily orally in three divided doses and, one week later, a bolus injection of VCR was administered at full dose. Three days later she developed an intermittent hoarse voice lasting about 72 hours. Moreover, she manifested reduction of deep tendon reflexes, neuritic calf pain (treated with oral acetaminophen) and foot drop; the pain and foot drop resolved after about 24 hours. The following weekly VCR was omitted and glutamic acid was stopped after 10 days of overall treatment. The fourth and last dose of VCR was administered again at 1 mg/m^2^ and was well tolerated. She is now in complete remission and on maintenance therapy.

## Discussion

VCP appears in an early report on the use of VCR, but it seems to be very rare; in a recent review the prevalence among children receiving VCR was 1.36% [1]. Analyzing the literature, we found 18 cases of children with VCR-induced VCP (Table 1) and our patient is the fifth girl reported [1-9].

**Table 1:** Literature review of pediatric patients with vincristine induced vocal cord palsy

**Authors**	**Sex/age**	**Diagnosis**	**Duration, weeks**	**Vocal cord palsy**	**Airway intervention**	**Swallowing dysfunction**	**Associated neuropathy**	**Subsequent vincristine administration**
Tobias and Bozeman [2]	M/5 years	ALL	0.5	L	No	No	No	Omitted
	F/2 years	NHL-T	2	L	No	No	Yes	Half dose
Annino *et al*. [3]	M/1.5 years	RMS	6	BL	No	Yes	No	Omitted
	M/1.3 years	Ependymoma	2	BL	No	No	No	Omitted
	M/3 years	ALL	2	L	No	No	Yes	Half dose
Agarwall *et al*. [4]	F/11 years	LNH	NA	R	No	No	No	NA
Anghelescu *et al*. [5]	NA/0.4 years	ALL	1	BL	Yes	Yes	No	NA
Graf *et al*. [6]	M/15 years	ALL + CMT	34.3	BL	No	No	Yes	NA
Ahmad *et al*. [7]	M/3 years	ALL + Down’s	38.5	BL	Yes	Yes	No	Omitted
	M/1.8 years	Ependymoma	34.3	BL	No	No	No	Omitted
	M/2 years	ALL	27	BL	No	Yes	Yes	Omitted*
Kuruvilla *et al*. [1]	F/1 year	ALL	37	BL	Yes	Yes	No	Omitted
	M/4 years	ALL + Down’s	20	BL	Yes	No	Yes	Half dose
	M/5 years	ES	7	BL	No	No	No	No indication
	M/3 years	RMS	4	L	No	Yes		Half dose, then increased to regular regimen
Latiff *et al*. [8]	M/2 years	ALL	13	BL	No	No	No	Half dose**
	F/3 years	ALL	40	BL	Yes***	No	No	Half dose
Naithani *et al*. [9]	M/14 years	ALL	6	BL	No	Yes	Yes	Omitted

*Vincristine definitely omitted after symptoms recurrence in consequence of drug reintroduction at full dose (drug initially given at 25% of recommended dose and gradually increased to full dose)

**Vincristine definitely omitted after symptoms recurrence in consequence of drug reintroduction at half dose

***Cordectomy.

ALL = acute lymphoblastic leukemia; BL = bilateral; CMT = Charcot Marie Tooth; Down’s = Down’s syndrome; ES = Ewing’s sarcoma; L = left; NA = not assessed; NHL-T = T cell non-Hodgkin’s lymphoma; R = right; RMS = rhabdomyosarcoma

Most cases are seen in younger children, with cases also reported in babies [5]; the median age is 2.7 years and 12 out of 18 patients were less than three years of age. The paralysis can be bilateral or, in about one-quarter of cases, unilateral, with the left vocal cord seemingly much more commonly affected (four cases versus one case of right VCP). Swallowing problems are fairly common (about one-third of the patients) and, apparently only when the paralysis is bilateral, a period of mechanical ventilation may be required. Remarkably, assisted ventilation was necessary only in five out of 18 patients [1,5,7,8]. It is fairly certain that Down’s syndrome patients are at higher risk of airway intervention (two out of two reported cases) [1,7]. The laryngeal dysfunction can be isolated or associated, as in about one-third of patients, with other manifestations of neurotoxicity, but never with other cranial neuropathies [1-3,6,7,9]. The problem resolves in a period of one to 10 months, usually with stridor resolving before the hoarseness. The median duration is six weeks.

The role of glutamic acid in decreasing VCR-induced neurotoxicity has been analyzed in some studies. Recently, a trial in a pediatric population [10] supported the efficacy of glutamic acid in preventing the neurological side effects of VCR via a mechanism that remains to be elucidated, but, to the best of our knowledge, it has never been used after first occurrence of neurotoxicity. In our patient glutamic acid, as rescue treatment, proved to be ineffective and when a full dose of VCR was given one week after a reduced dose, our patient showed not only a reappearance of stridor and hoarseness but also a transient peripheral neuropathy. On reviewing pediatric cases, reintroduction of VCR at half dose was associated with a recurrence of palsy in only one case out of six [8], and no respiratory failure developed. In contrast, besides our patient, there are only another two cases reported of VCR reintroduction at full dose [1,7], and in one of them there was a relapse [7].

## Conclusions

In our opinion the administration of weekly VCR at half to two-thirds dose should be the recommended initial approach after a case of drug-induced vocal cord palsy. We have not found evidence that administration of glutamic acid can prevent relapse of VCP.

## Consent

Written informed consent was obtained from the parents of the patient for publication of this case report and any accompanying images. A copy of the written consent is available for review by the Editor-in-Chief of this journal.

## Competing interests

The authors declare that they have no competing interests.

## Authors’ contributions

PF made substantial contributions to the structure of the manuscript, was involved in clinical management of our patient and drafted the manuscript. ST was involved in clinical management and helped draft the manuscript. SC and GB analyzed and interpreted patient data regarding disease. GO was involved in pharmaceutical management of the disease, and participated in drafting part of the manuscript. PD made substantial contributions to the structure of the manuscript, was involved in clinical management of our patient, and was a major contributor in writing the manuscript. All authors read and approved the final manuscript.
